# Racial and ethnic disparities in statin adherence: insights from the All of Us Research Program

**DOI:** 10.3389/fcvm.2025.1541082

**Published:** 2025-12-12

**Authors:** Gabriela Escobar, Zahra Azizi, Anne de Hond, Ashley Adanna Lewis, Madelena Y. Ng, Fatima Rodriguez, Tina Hernandez-Boussard

**Affiliations:** 1 Stanford University, Stanford, CA, United States; 2 Division of Cardiovascular Medicine, Stanford University, Stanford, CA, United States; 3 Julius Center for Health Sciences and Primary Care, University Medical Center Utrecht, Utrecht, Netherlands; 4 Department of Biomedical Data Science, Stanford University, Stanford, CA, United States; 5 Center for Digital Health, Stanford University, Stanford, CA, United States

**Keywords:** statin, adherence, SDoH, race and ethnicity, All of Us cohort

## Abstract

**Background:**

Statin adherence impacts cardiovascular outcomes, yet disparities persist. Understanding the sociodemographic factors and barriers is crucial for targeted interventions.

**Objective:**

To investigate the relationship between sociodemographic factors and statin adherence across racial and ethnic groups.

**Design:**

This retrospective study examined sociodemographic factors, prescription records, clinical factors, and responses from the Demographic, Drug Exposure, Healthcare Utilization Survey (HUS) in the All of Us (AoU) cohort. Multivariable logistic regression models were used to assess the impact of sociodemographic factors on adherence stratified by race.

**Participants:**

Adult participants with statin prescription records. Subgroup analyses included those who responded to the HUS.

**Exposures:**

Statin prescription.

**Main outcomes and measures:**

We calculated percent days covered (PDC) as the proportion of days within a year in which a person prescribed a statin filled a prescription. Adequate adherence was defined as PDC ≥ 80%.

**Results:**

Among the 17,029 adults with a statin prescription, the mean PDC was 57%, and 66% had PDC ≤ 80%. In multivariable analyses stratified by race and ethnicity, distinct barriers to adherence emerged. Among the non-Hispanic White participants, barriers to consistent healthcare [odds ratio (OR) = 0.60, 95% CI (0.42–0.87)] and lack of provider identity concordance [OR = 0.83, 95% CI (0.72–0.97)] were associated with lower adherence. In the non-Hispanic Black participants, Medicare [OR = 0.54, 95% CI (0.32–0.90)] and Veterans Affairs insurance [OR = 0.44, 95% CI (0.20–0.96)], as well as financial barriers [OR = 0.69, 95% CI (0.51–0.96)], reduced adherence. Among the Hispanic participants, provider-related anxiety [OR = 0.13, 95% CI (0.02–0.87)], immigrant status [OR = 0.25, 95% CI (0.08–0.72)], and Medicaid coverage [OR = 0.11, 95% CI (0.03–0.45)] predicted lower adherence.

**Conclusions and relevance:**

Addressing cardiovascular disease disparities requires recognizing unique sociodemographic barriers to statin adherence within racial and ethnic groups. Our findings highlight the need for tailored strategies considering the diverse barriers each group faces. Targeted interventions can bridge adherence gaps and improve cardiovascular outcomes across populations. This approach recognizes that although race and ethnicity may correlate with specific barriers, the underlying social determinants of health often play the key role in statin adherence.

## Introduction

Atherosclerotic cardiovascular disease (ASCVD) remains the leading cause of death and disability globally. In the United States, ASCVD accounts for one in three deaths, causing approximately 800,000 deaths each year (1). Statins are the first-line therapy for ASCVD prevention and have been shown to significantly reduce cardiovascular morbidity and mortality (2). However, adherence to statin therapy, despite its clinical efficacy, remains suboptimal and inequitable—nearly half of adults discontinue statin therapy within 1 year, and non-adherence has been associated with an increased risk of recurrent cardiovascular events and premature mortality (3).

Growing evidence shows that statin non-adherence is shaped by social and structural inequities rather than individual behavior alone. Women and racial and ethnic minority groups, particularly Black and Hispanic adults, have consistently lower adherence than White adults (3, 4). The social determinants of health (SDoH)—including socioeconomic disadvantage, lack of insurance, unstable employment, and limited access to care—are strongly associated with early discontinuation (5, 6). Structural barriers, such as cost-related medication non-adherence, medical mistrust, and inequities in prescribing and follow-up, contribute to these disparities. These system-level drivers often reflect the effects of structural racism embedded in clinical practices and access pathways, including provider bias resulting in the undertreatment of high-risk Black and Hispanic patients (7–10). Despite consistent evidence of inequity, most of the previous studies rely on claims or health system data that lack meaningful SDoH measures, such as discrimination, financial strain, caregiving burden, and housing instability, limiting our ability to understand the mechanisms of non-adherence (11).

The NIH All of Us (AoU) Research Program (AoURP) presents a unique opportunity to address this knowledge gap through its integration of electronic health records (EHRs) with detailed, participant-reported social and behavioral data in an intentionally diverse national cohort (12). Guided by prior evidence, we hypothesize that (1) statin adherence will be lower among Black and Hispanic adults compared with White adults and (2) adverse social determinants of health—such as low income, lack of insurance, and social or economic adversity—will be associated with lower adherence and partially explain racial and ethnic disparities. Therefore, the objectives of this study are (1) to characterize disparities in statin adherence across diverse population subgroups in the All of Us cohort and (2) to evaluate the social and structural factors associated with adherence patterns. These insights will support the development of targeted, equity-focused interventions that promote sustained statin use and improve cardiovascular outcomes in at-risk communities.

## Methods

In this study, we perform a retrospective analysis to first assess how sociodemographic factors relate to statin adherence and second to characterize reasons for non-adherence utilizing the AoU cohort. This study was approved by the Institutional Review Board of Stanford University.

### Data source

We used the All of Us (AoU) v6 Controlled Tier dataset, a multi-domain database from the NIH's Precision Medicine Initiative (13). The dataset integrates EHRs, physical measurements, biospecimens, and participant surveys. We analyzed three domains in the AoU dataset, namely, the demographic, drug exposure, and healthcare utilization survey (HUS) domains.

### Study population

The study cohort included individuals aged 18 or older who were prescribed a statin medication between 2017 and 2020, following the AoU v6 Controlled Tier data cutoff (Overall Cohort). Statin prescriptions were identified using RxNorm codes, which included all generic and brand-name statins (Supplementary Table S1). To ensure that our study cohort and respective biometrics and survey responses represented the participants accurately, we included only individuals with a statin prescription documentation within 1 year at the time of enrollment. Participants with records that lack days supplied values or with suppressed information were excluded from the analysis. Participants were also excluded if their race was documented as “Asian” or “other: American Indian/Alaskan Native, Middle Eastern, Asian, Native Hawaiian/Pacific Islander” and sex at birth as “other” due to the small sample sizes of these groups. We established a subset of individuals who responded to the HUS to analyze adherence factors across racial and ethnic subgroups (Survey Cohort). We defined our survey cohort as participants who responded to the HUS question “Took Less Medicine to Save Money”, the survey question with the highest response rate.

### Participant characteristics

Participants' data were collected at the time of enrollment into the AoU cohort. The Demographic domain included date of birth, sex at birth, and self-reported race, ethnicity, and gender identities. Age was calculated as the time elapsed from the date of birth to the time of enrollment. Race and ethnicity were categorized as Hispanic, non-Hispanic White (NHW), and non-Hispanic Black (NHB). Sex at birth was self-reported (e.g., male and female). Other demographic variables, including insurance status, socioeconomic status, educational attainment, and immigration status, were based on self-reported demographic data.

Statin prescriptions were captured from the drug exposure domain, including fields on the number of days supplied, prescription start dates, and prescription names. Comorbidities such as myocardial infarction, liver disease, and diabetes were identified using the International Classification of Diseases (ICD)-9 and ICD-10 codes. The Charlson comorbidity index was calculated from EHRs. The mean values of lab measurements for high-density lipoprotein cholesterol (HDL-C) and low-density lipoprotein cholesterol (LDL-C) levels and serum creatinine 12 months prior to the index date were included. Vital signs were obtained at the index date (see Supplementary Table S1 for data retrieval codes).

The HUS domain comprises qualitative responses of participants about healthcare engagements. Questions evaluated the impact of access, utilization, and quality on individuals' healthcare experiences and subsequent outcomes (10). Responses included 10 categories from the HUS, with thematically similar questions collapsed into survey variables (Supplementary Table S2). The patients were subsequently categorized based on reported barriers. If they experienced any barrier within a category, the variable was coded as *true*. If data were missing across the entire composite category, it was coded as *unknown*. Otherwise, it was coded as *false*.

### Outcome

The main outcome was percent days covered (PDC), calculated as the proportion of days in a 365-day period that an individual with an active statin prescription obtained the prescription at the required dose. It is an established measure of adherence that approximates treatment access and use (14). In this study, PDC was determined using the “days supplied” value from prescription records. Adequate adherence was defined as PDC ≥ 0.80, based on the established criteria from previous studies. Ensuring that adherence estimates reflected current use required participants to have at least one statin prescription or fill within the prior year; those without a prescription during this window were excluded, consistent with PDC's definition as a measure of adherence among active users. The time of study consent served as the index date, aligning adherence calculations with recent participant data and survey questions that inquired about behavioral experiences in the prior year, thereby improving temporal correspondence between self-reported barriers and observed medication behavior.

### Statistical analysis

The study was conducted in two phases. The first phase evaluated the association between demographics and statin adherence in the overall cohort using chi-squared tests and Student's *t*-tests. Univariate and multivariate regression models examined the association between sociodemographic factors and adequate adherence, adjusting for age, sex, and clinical characteristics.

The second phase involved examining reasons for statin adequate adherence compared with non-adherence (PDC < 0.80%) in participants who completed the HUS. The cohort was stratified by racial and ethnic background to identify race and ethnic-specific reasons for non-adherence. Stepwise logistic regression modeling was used to identify predictors affecting statin adherence within each racial and ethnic subgroup. Sociodemographic and survey variables were included, and the model was optimized for the Akaike information criterion. Model fit was assessed using the adjusted *R*^2^, with a variable entry at *p* < 0.05 and removal at *p* > 0.10 to prevent overfitting. Respondents with unknown barrier categories were retained in the cohort to account for missing data, while patients with missing clinical covariates required for the logistic regression models were excluded from the analytic sample. Models were compared across subgroups to identify the differences in predictive profiles. All analyses were conducted on the AoURP platform using Python and R Workbench, with statistical significance defined as *p* < 0.05.

## Results

### Baseline cohort

**Figure 1:**
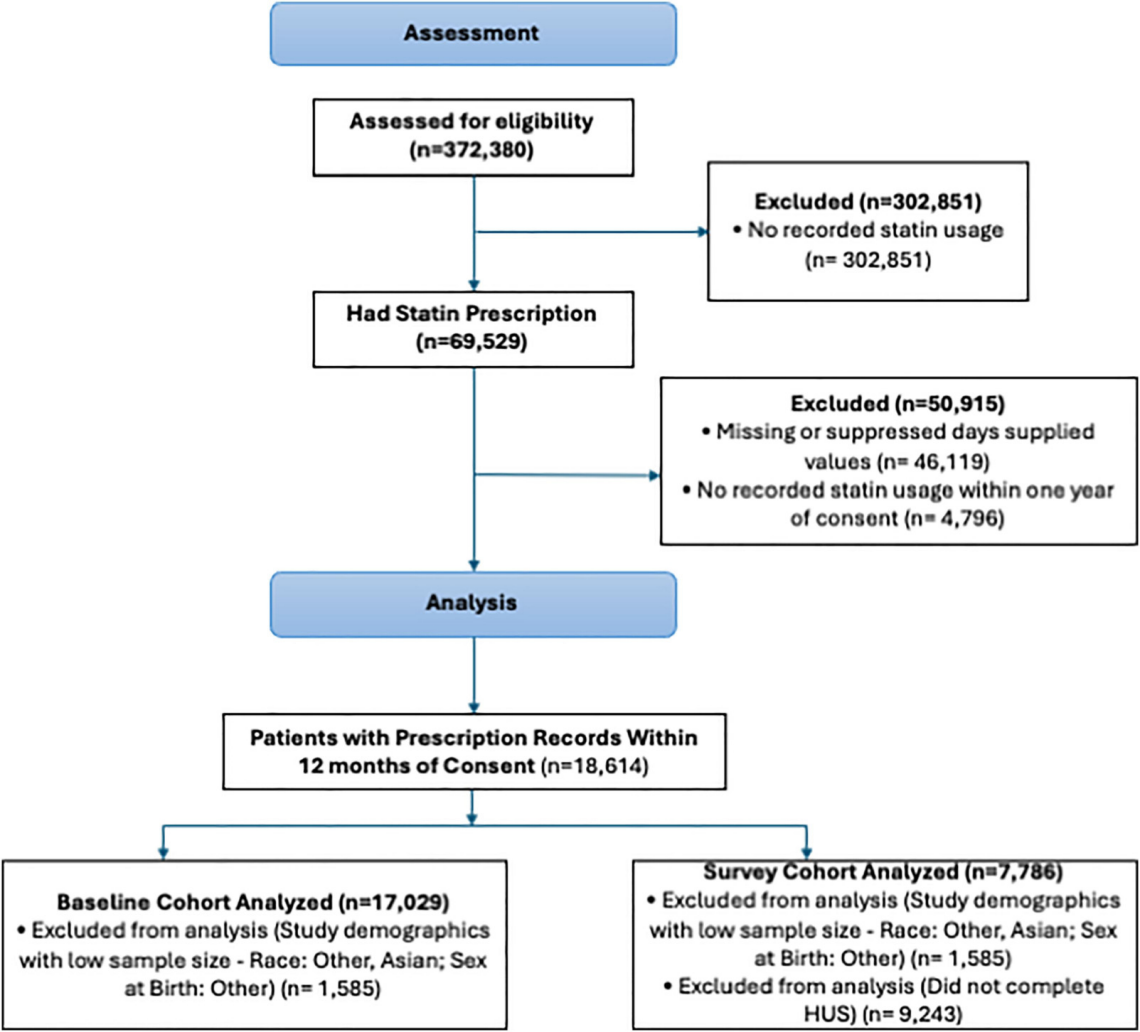
Study consort diagram. The exclusion criteria for the primary study population depended on differential data availability across study participants.

From a cohort of 372,380 participants, 69,529 individuals were prescribed statins (Figure 1). After applying inclusion and exclusion criteria—which excluded participants with missing or suppressed days supplied (46,119), those with no recorded usage within 1 year of consent (4,796), and study demographics with low sample sizes including other sex, other race, and Asian (1,585)—17,029 participants with active statin prescriptions at the time of enrollment were included in the analysis. Of these, 7,786 completed the HUS (survey cohort). The mean age was 64.4 years (SD 11.5), and 50.2% were female at birth. Over two-thirds of the participants (67.1%) were non-Hispanic White, 21.6% were non-Hispanic Black, and 11.3% were Hispanic. Foreign-born participants accounted for 9.4% of the cohort (Table 1).

**Table 1:** Characteristics of the baseline and survey cohorts.

Variable	Baseline cohort	Survey cohort	*p*-value
*n*	17,029	7,786	
Percent days covered [mean (SD)]	0.58 (0.35)	0.64 (0.33)	<0.001
Age [mean (SD)]	64.39 (11.47)	64.92 (10.80)	0.001
Sex at birth			0.98
Male	8,475 (49.8)	3,877 (49.8)	
Female	8,554 (50.2)	3,909 (50.2)	
Race/ethnicity			<0.001
Non-Hispanic White	11,426 (67.1)	6,337 (81.4)	
Non-Hispanic Black	3,685 (21.6)	1,057 (13.6)	
Hispanic	1,918 (11.3)	392 (5.0)	
Insurance status			<0.001
Private	2,758 (16.2)	1,610 (20.7)	
Medicaid	3,535 (20.8)	914 (11.7)	
Medicare	4,875 (28.6)	2,546 (32.7)	
Other	876 (5.1)	389 (5.0)	
VA	1,860 (10.9)	866 (11.1)	
Unknown	3,125 (18.4)	1,461 (18.8)	
Education			<0.001
Post-secondary	11,606 (68.2)	6,284 (80.7)	
Secondary	4,612 (27.1)	1,332 (17.1)	
Less than secondary	521 (3.1)	104 (1.3)	
Unknown	290 (1.7)	66 (0.8)	
Income status			<0.001
High >$75,000	3,859 (22.7)	2,582 (33.2)	
Middle $35,000–75,000	3,771 (22.1)	2,154 (27.7)	
Low <$35,000	6,636 (39.0)	2,222 (28.5)	
Unknown	2,763 (16.2)	828 (10.6)	
Immigrant status			<0.001
US-born	15,427 (90.6)	7,384 (94.8)	
Foreign-born	1,602 (9.4)	402 (5.2)	
Employment status			<0.001
Employed	5,306 (31.2)	2,646 (34.0)	
Not employed	11,723 (68.8)	5,140 (66.0)	
Marital status (%)			<0.001
Single	2,573 (15.1)	952 (12.2)	
Married/widowed	8,444 (49.6)	4,563 (58.6)	
Separated/divorce	5,728 (33.6)	2,195 (28.2)	
Unknown	284 (1.7)	76 (1.0)	
Smoking			<0.001
Never	8,070 (47.4)	3,933 (50.5)	
Current	2,606 (15.3)	759 (9.7)	
Former	5,907 (34.7)	2,993 (38.4)	
Unknown	446 (2.6)	101 (1.3)	
CCI			<0.001
Low	3,302 (19.4)	1,797 (23.1)	
High	13,727 (80.6)	5,989 (76.9)	
Serum creatinine [mean (SD)]	2.74 (3.75)	2.24 (3.35)	<0.001
SBP [mean (SD)]	132.17 (17.82)	131.03 (16.46)	<0.001
HDL [mean (SD)]	37.54 (16.12)	38.74 (16.05)	<0.001
LDL [mean (SD)]	84.81 (33.30)	84.76 (32.05)	0.931
Total chol. [mean (SD)]	170.96 (42.35)	171.58 (40.15)	0.362
DBP [mean (SD)]	78.26 (11.04)	77.33 (10.15)	<0.001
BMI [mean (SD)]	31.83 (7.26)	31.72 (7.15)	0.277
Myocardial infarction (%)	2,540 (14.9)	817 (10.5)	<0.001
CHF (%)	3,398 (20.0)	1,044 (13.4)	<0.001
Liver disease (%)	3,361 (19.7)	1,290 (16.6)	<0.001
Any diabetes (%)	7,510 (44.1)	2,801 (36.0)	<0.001

Continuous variables are presented as mean ± standard deviation (SD) or median (IQR), as appropriate. Categorical variables are reported as counts and percentages based on all responses, with “unknown” included as a separate category and included in percentage calculations to ensure transparency in data completeness. *p*-values reflect comparisons between the overall cohort and the survey cohort using chi-squared tests for categorical variables and *t*-tests or Wilcoxon rank-sum tests for continuous variables. Denominators for percentages are based on the total number of participants in each cohort.

Other race category includes American Indian/Alaskan native, Middle Eastern, Asian, and Native Hawaiian/Pacific Islander.

SD, standard deviation; SBP, systolic blood pressure; HDL, high-density lipoprotein; LDL, low-density lipoprotein; chol, cholesterol; BMI, body mass index; MIP, myocardial infarction; CHF, congestive heart failure.

### Statin prescriptions

**Figure 2:**
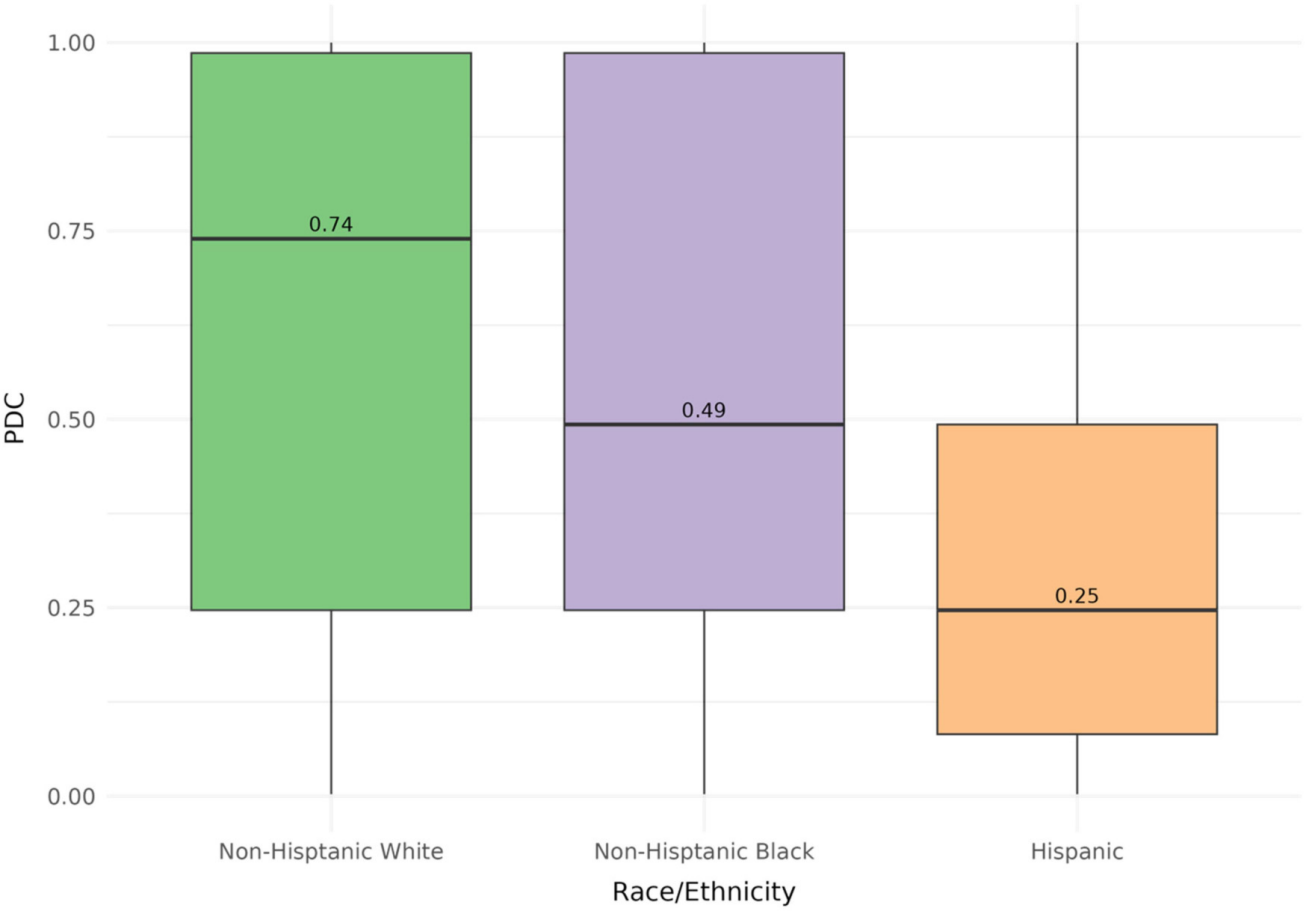
Distribution of PDC stratified by racial and ethnic subgroups. Stratified analysis on the baseline cohort (*n* = 17,029) revealed differences in median PDC (NHW, 0.740; NHB, 0.493; Hispanics, 0.247) when comparing racial and ethnic subgroups.

Among the 17,029 participants, the mean statin PDC was 58%, with 66% reporting a PDC ≤ 80% (Table 2). Non-adherent participants were younger (63.4 ± 11.7 years vs. 66.2 ± 10.7 years), were more likely to be female (51.3% vs. 48.3%), had lower comorbidity burden (78.8% vs. 84.1% with high comorbidities), and included a higher proportion of foreign-born individuals (12.1% vs. 4.4%) than adherent participants, as well as NHB (22.3% vs. 20.4%) or Hispanic (15.2% vs. 3.8%) participants than adherent participants. When the cohort was stratified by racial and ethnic groups, NHW participants had a median PDC of 74% [interquartile range (IQR) (0.25–0.98)], Black participants had 49% [IQR (0.25–0.98)], and Hispanic participants had 25% [IQR (0.08–0.49)] (Figure 2).

**Table 2:** Bivariate logistic regression analysis of the baseline cohort according to percent days covered (PDC) of statin prescription.

Outcome: PDC ≥ 0.80	Overall baseline cohort		
	PDC < 0.80 non-adherent	PDC ≥ 0.80 adherent	Odds ratio (95% CI)
*n*	11,178	5,851	
Percent days covered [mean (SD)]	0.37 (0.24)	0.98 (0.04)	
Age [mean (SD)]	63.43 (11.73)	66.22 (10.70)	1.02 (1.02–1.03)
Sex at birth (%)			
Male	5,449 (48.7)	3,026 (51.7)	Reference
Female	5,729 (51.3)	2,825 (48.3)	0.89 (0.83–0.95)
Race/ethnicity (%)			
Non-Hispanic White	6,993 (62.6)	4,433 (75.8)	Reference
Non-Hispanic Black	2,491 (22.3)	1,194 (20.4)	0.76 (0.7–0.82)
Hispanic	1,694 (15.2)	224 (3.8)	0.21 (0.18–0.24)
Insurance status (%)			
Private	1,835 (16.4)	923 (15.8)	Reference
Medicaid	2,559 (22.9)	976 (16.7)	0.77 (0.7–0.84)
Medicare	3,057 (27.3)	1,818 (31.1)	1.22 (1.12–1.33)
Other	599 (5.4)	277 (4.7)	0.88 (0.76–1.02)
VA	1,075 (9.6)	785 (13.4)	1.43 (1.28–1.59)
Unknown	2,053 (18.4)	1,072 (18.3)	-
Education (%)			
Post-secondary	7,394 (66.1)	4,212 (72.0)	Reference
Secondary	3,148 (28.2)	1,464 (25.0)	0.82 (0.76–0.88)
Less than secondary	443 (4.0)	78 (1.3)	0.31 (0.25–0.4)
Unknown	193 (1.7)	97 (1.7)	-
Income status (%)			
High >$75,000	2,426 (21.7)	1,433 (24.5)	Reference
Middle $35,000–75,000	2,318 (20.7)	1,453 (24.8)	1.03 (0.95–1.12)
Low <$35,000	4,518 (40.4)	2,118 (36.2)	0.75 (0.69–0.81)
Unknown	1,916 (17.1)	847 (14.5)	-
Immigrant status (%)			
US-born	9,831 (87.9)	5,596 (95.6)	Reference
Foreign-born	1,347 (12.1)	255 (4.4)	0.33 (0.29–0.38)
Employment status (%)			
Employed	3,659 (32.7)	1,647 (28.1)	Reference
Not employed	7,519 (67.3)	4,204 (71.9)	1.24 (1.16–1.33)
Marital status (%)			
Single	1,730 (15.5)	843 (14.4)	Reference
Married/widowed	5,396 (48.3)	3,048 (52.1)	1.17 (1.07–1.28)
Separated/divorce	3,858 (34.5)	1,870 (32.0)	1.01 (0.91–1.11)
Unknown	194 (1.7)	90 (1.5)	-
Smoking (%)			
Never	5,349 (47.9)	2,721 (46.5)	Reference
Current	1,852 (16.6)	754 (12.9)	0.8 (0.72–0.87)
Former	3,670 (32.8)	2,237 (38.2)	1.19 (1.11–1.28)
Unknown	307 (2.7)	139 (2.4)	-
Charlson comorbidity index (%)			
Low	2,373 (21.2)	929 (15.9)	Reference
High	8,805 (78.8)	4,922 (84.1)	1.43 (1.31–1.55)
Serum creatinine [mean (SD)]	3.15 (4.08)	2.00 (2.91)	0.9 (0.89–0.91)
SBP [mean (SD)]	132.31 (18.05)	131.89 (17.37)	1 (1–1)
HDL [mean (SD)]	36.97 (16.68)	38.45 (15.15)	1.01 (1–1.01)
LDL [mean (SD)]	88.86 (35.17)	78.40 (28.98)	0.99 (0.99–0.99)
Total chol. [mean (SD)]	175.11 (44.82)	164.48 (37.24)	0.99 (0.99–0.99)
DBP [mean (SD)]	78.88 (11.25)	77.08 (10.52)	0.99 (0.99–0.99)
BMI [mean (SD)]	31.82 (7.35)	31.84 (7.08)	0.98 (0.98–0.98)
Myocardial infarction (%)	1,710 (15.3)	830 (14.2)	0.92 (0.84–1)
Liver disease (%)	2,285 (20.4)	1,076 (18.4)	0.46 (0.44–0.50)
Any diabetes (%)	4,933 (44.1)	2,577 (44.0)	0.52 (0.50–0.55)

Analysis conducted on the baseline cohort (*n* = 17,029). Continuous variables are presented as mean ± standard deviation (SD) or median (IQR), as appropriate. Categorical variables are reported as counts and percentages based on all responses, with “unknown” included as a separate category to ensure transparency in data completeness. Unadjusted ORs with 95% CIs are reported for each variable. ORs for continuous variables represent the change in odds per one-unit increase in the corresponding measure.

Reference categories are as follows: non-Hispanic White for race, private insurance for insurance status, post-secondary education for educational attainment, high income for income level, US-born for immigrant status, single for marital status, employed for employment status, never smoker for smoking status, and low comorbidity for Charlson comorbidity index.

Other race category includes American Indian/Alaskan native, Middle Eastern, Asian, and Native Hawaiian/Pacific Islander.

PDC, percent days covered; CI, confidence interval; SD, standard deviation; CCI, Charlson comorbidity index; SBP, systolic blood pressure; HDL, high-density lipoprotein; LDL, low-density lipoprotein; chol, cholesterol; DBP, diastolic blood pressure; BMI, body mass index; MI, myocardial infarction; CHF, congestive heart failure.

### Baseline cohort analysis

**Figure 3:**
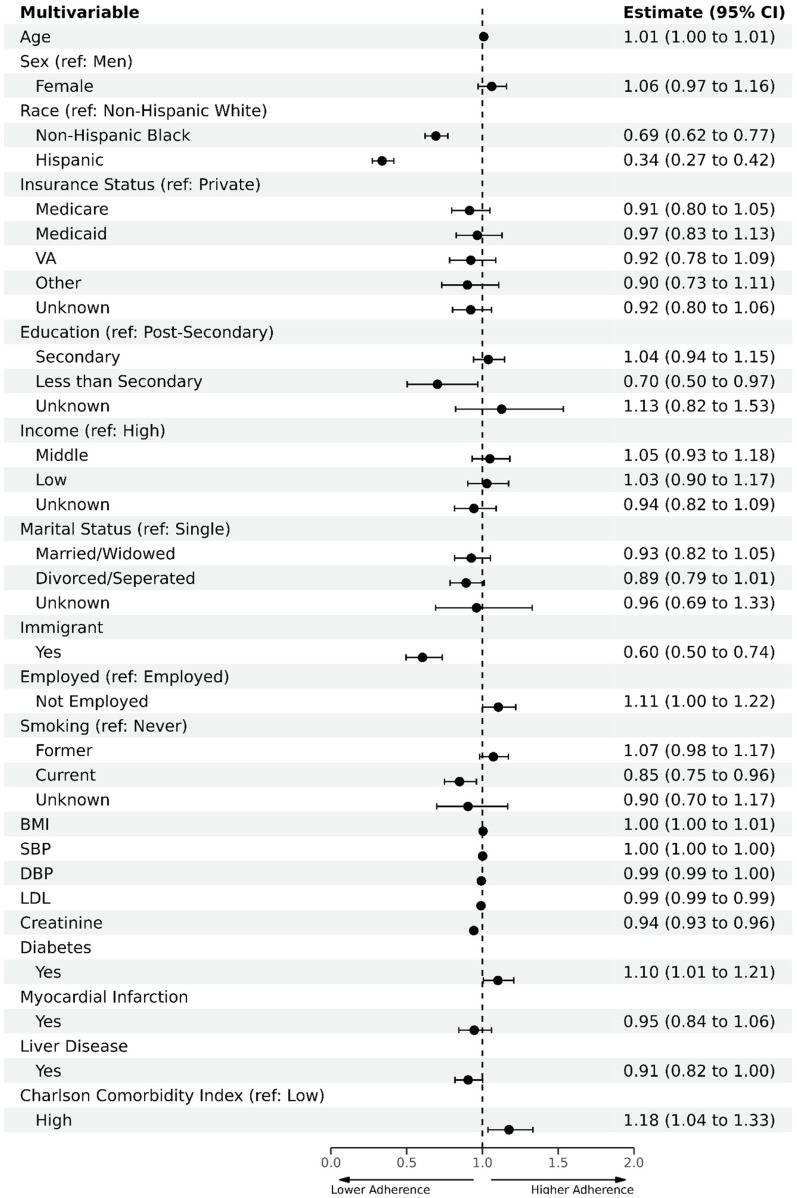
Multivariate logistic regression analysis to evaluate factors associated with statin adherence in baseline cohort. Primary analysis on the baseline cohort (*n* = 17,029) included all baseline sociodemographic variables and was adjusted for clinical variables, including SBP, DBP, and LDL. Reference categories are as follows: non-Hispanic White for race, private insurance for insurance status, post-secondary education for educational attainment, high income for income level, US-born for immigrant status, single for marital status, employed for employment status, never smoker for smoking status, and low comorbidity for Charlson comorbidity index.

The multivariable regression model identified factors associated with adequate statin adherence (Figure 3). Higher adherence was associated with having diabetes [odds ratio (OR) = 1.10, 95% CI (1.01–1.21)] or a Charlson index of 3 or higher [OR = 1.18, 95% CI (1.04–1.33)]. Lower adherence was observed among NHB [OR = 0.69, 95% CI (0.62–0.77)], Hispanics [OR = 0.33, 95% CI (0.27–0.42)], and immigrants [OR =  0.60, 95% CI (0.50–0.74)] compared with NHWs and US-born individuals.

### Survey cohort analysis

In the sub-cohort of 7,786 participants who completed the HUS, the mean statin PDC was 0.64, and 60.8% had low adherence. The mean age of this sub-cohort was 64.9 years (SD: 10.80), and 81.4% were non-Hispanic White, 13.6% non-Hispanic Black, and 5.0% Hispanic. Immigrant participants constituted 5.2% of the cohort. Survey participants were predominantly NHW, had a smaller share of Medicare or Medicaid insured individuals, and exhibited higher education and income levels compared with the overall cohort (Table 1). Non-adherent survey participants shared characteristics with the overall non-adherent cohort, including more females, younger, Hispanic or NHB, lower income and education levels, immigrant status, and non-employed (Supplementary Table S3).

Multivariable stepwise analyses revealed distinct barriers to statin adherence within each racial and ethnic group when stratified by race and ethnicity (Table 3). Among NHW participants, experiencing barriers to consistent healthcare [OR = 0.60, 95% CI (0.42–0.87)] and limited access to providers with shared identities [OR = 0.83, 95% CI (0.72–0.97)] were significantly associated with lower adherence. Higher adherence was associated with unemployment [OR = 1.2, 95% CI (1.0–1.4)]. For NHB participants, Medicare [OR = 0.54, 95% CI (0.32–0.90)] and Veterans Affairs (VA) insurance [OR = 0.44, 95% CI (0.20–0.96)] types were associated with lower adherence compared with private insurance. Financial barriers [OR = 0.69, 95% CI (0.51–0.96)] were also associated with lower adherence. Lower educational attainment, specifically having secondary education or less, was associated with higher adherence compared with post-secondary education [OR = 1.44, 95% CI (1.04–2.00)]. Among the Hispanic participants, anxiety about seeing a healthcare provider [OR = 0.13, 95% CI (0.02–0.87)] and immigrant status [OR = 0.25, 95% CI (0.08–0.72)] were independently associated with lower odds of adherence. Medicaid coverage compared with private insurance also predicted non-adherence [OR = 0.11, 95% CI (0.03–0.45)].

**Table 3:** Multivariable stepwise logistic regression to assess the barriers for Non-adherence based on survey results.

Multivariable stepwise	OR (95% CI)	OR (95% CI)	OR (95% CI)
**Logistic regression outcome: PDC ≥ 0.80**	**Non-Hispanic White**	**Non-Hispanic Black**	**Hispanic**
Survey variables			
Difficulty accessing concordant provider	**0.83 (0.72–0.97)** ^a^		0.92 (0.15–5.79)
Healthcare inaccessibility	**0.60 (0.42–0.87)^a^**		
Financial barriers		**0.69** (**0.51–0.94)^a^**	
Patient anxiety			**0.14** (**0.02–0.6)^a^**
Social dependency barriers		0.77 (0.25–2.34)	**0.53** (**0.01–29.99)^a^**
Transportation barrier		0.91 (0.59–1.41)	
Social demographic variables			
Sex at birth (ref: Male)			
Female	1.12 (0.99–1.27)		
Insurance (ref: Private)			
Medicare		**0.54** (**0.32–0.90)****^a^**	0.38 (0.06–2.25)
Medicaid		0.99 (0.62–1.59)	**0.11** (**0.03–0.44)^a^**
VA		**0.44** (**0.20–0.96)****^a^**	0.31 (0.08–1.20)
Other		0.44 (0.19–1.02)	0.82 (0.15–4.51)
Unknown		0.76 (0.47–1.23)	**0.21** (**0.05–0.83)****^a^**
Education (ref: Post-Secondary)			
Less than secondary		**2.29** (**1.01–5.18)^a^**	
Secondary		**1.44** (**1.04–2.00)^a^**	
Unknown		0.74 (0.16–3.30)	
Immigrant			**0.25** (**0.09–0.72)^a^**
Employment (ref: Employed)			
Not employed	1.19 (1.05–1.35)		

Analysis conducted on the survey cohort (*n* = 7,786). The input in the stepwise logistic regression model included all sociodemographic factors and comorbidities in addition to survey responses in each racial and ethnic subgroup. The stepwise model is adjusted for age and sex across all strata. OR <1 indicates less likely to be adherent, whereas OR >1 indicates more likely to be adherent.

Bolded values highlight statistically significant results.

Other race categories include American Indian/Alaskan Native, Middle Eastern, Asian, and Native Hawaiian/Pacific Islander.

VA, Veterans Affairs; PDC, Percent Days Covered; CI, Confidence Interval.

a Statistically significant (*p* < 0.05) results.

## Discussion

This study using the AoU research cohort revealed low adherence to statin medications, with over two-thirds of the participants reporting adherence rates of 80% or less. Adherence was lower among racial and ethnic minorities. The key factors associated with adherence included employment status, comorbidity index, and immigration status, with the lowest adherence rates among NHB, Hispanic, and immigrant participants. We identified distinct clusters of SDoH factors that varied significantly across racial and ethnic groups and were associated with differences in adherence. This finding underscores complex and nuanced interactions between social and structural factors that affect health outcomes.

In examining statin adherence across different racial and ethnic groups within the AoU cohort, certain commonalities emerged. Socioeconomic status, measured by insurance type and income level, was a shared determinant across all groups, a finding emphasized in separate studies advocating for the reduction of costs through insurance coverage or regulatory measures to improve medication adherence (5, 6, 10). Patients with government-sponsored healthcare plans (e.g., Medicare or Veterans Health) had lower adherence rates than those of patients without insurance, suggesting unmeasured barriers to adherence among this predominantly older population with higher chronic disease burden (15–17).

For NHW participants, distinct factors influenced statin adherence that differed from other racial and ethnic minorities. Reduced access to consistent healthcare and limited availability of providers who shared patients' identities emerged as significant barriers to adherence. This suggests that, even among NHW individuals who are racially represented in the healthcare work, relational aspects of care—such as feeling understood, valued, and culturally aligned—may play an important role in treatment engagement. The finding challenges the assumption that provider–patient identity concordance is primarily relevant only to racial and ethnic minority populations (18). Instead, it underscores that trust and perceived connection to the healthcare system are broadly relevant across groups (19). Improving adherence among NHW individuals may therefore require strategies that expand access to continuous care while strengthening patient–provider relationships, such as increasing workforce diversity, enhancing cultural humility training, and investing in primary care continuity models that foster long-term trust (20).

Among the NHB participants, financial barriers were associated with significantly lower odds of statin adherence, with approximately 31% reduced likelihood of adherence compared with those without financial barriers. Similarly, reliance on safety-net coverage—specifically Medicare and VA insurance—was also negatively associated with adherence in this group. This finding suggests that the combined burden of healthcare costs and navigation of complex care systems may discourage continued medication use. Prior studies similarly link financial strain to medication underuse and delayed care among Black patients with ASCVD, patterns that are often compounded by structural inequities and social isolation (8, 21, 22). Recent national data indicate that one in eight ASCVD patients report cost-related barriers to statins, disproportionately affecting Black Americans, reinforcing that financial inaccessibility remains a major structural barrier to preventive cardiovascular therapy (22).

Notably, NHB participants with less than a secondary education demonstrated higher adherence compared with NHW participants. While this may reflect unmeasured protective factors—such as strong community support networks, culturally rooted health behaviors, or relational trust established with providers (23)—it may also reflect selection bias. NHB individuals with lower educational attainment who remain engaged in the healthcare system may represent a particularly motivated or health-literate subgroup (24, 25). Additionally, the relatively small size of the NHB subgroup in this analysis raises the possibility that this association may be unstable or due to chance. As such, this finding should be interpreted cautiously and examined further in larger and longitudinal studies.

For the Hispanic population within the AoU cohort, several factors impacted statin adherence. Immigration status was a significant predictor of adherence, with immigrants displaying notably lower adherence rates. Health literacy poses a major challenge for the broader US population but is particularly pronounced among immigrant communities who face complex healthcare systems, language barriers, and cultural differences (26–28). Medicaid insurance was also associated with lower adherence in this population, highlighting potential access gaps due to restricted eligibility requirements and complex case management. Lower adherence rates among immigrants may be partially due to the self-selection effect, where healthier and more optimistic individuals perceive non-life-threatening issues as less urgent (29). This is an important aspect to consider when comparing outcomes between other racial/ethnic groups. Public health innovations that strengthen bilingual communication, community-based support programs, and culturally competent healthcare providers can promote trust and confidence, enhancing adherence by addressing obstacles to continued treatment.

While this study provides meaningful insights, several limitations warrant consideration. First, the observed prevalence of statin prescriptions in our cohort appears lower than the national estimates (30, 31). This difference likely reflects both characteristics of the AoU population and incomplete medication capture (e.g., prescriptions written outside participating health systems or historical fills not recorded). Accordingly, our findings should not be interpreted as direct estimates of true statin use in the broader US population. Second, the survey-based HUS subset is subject to selection bias, overrepresenting non-Hispanic White, higher-income, and US-born individuals relative to the full cohort. As a result, barrier-specific estimates likely underrepresent the experiences of the most underserved populations. Estimates for certain subgroups, particularly Hispanic participants, should be interpreted with caution due to small event counts in some predictor categories, which contributed to wider CIs and reduced estimate stability. Moreover, small subgroup sizes (e.g., Asian and other racial/ethnic populations) precluded more comparative analyses. These limitations highlight the need for future studies to intentionally include a broader range of racial and ethnic subgroups to capture diverse experiences and better characterize disparities that may be obscured in aggregated analyses. Differences in key characteristics—including race, nativity, income, and education—between the survey cohort and the overall AoU population may further skew estimates of adherence barriers, particularly those rooted in structural disadvantage. This pattern of differential participation is consistent with longstanding literature showing lower response rates to voluntary research surveys among racial/ethnic minority and immigrant communities due to mistrust of research institutions, fears related to immigration status, prior experiences of discrimination, and historical misuse of data (32–35). Lastly, as an observational analysis involving multiple comparisons, this study cannot establish causality. While the associations observed offer valuable insights into potential mechanisms, some statistically significant findings may reflect random variation rather than true underlying effects and should therefore be interpreted with caution.

Despite these limitations, this work demonstrates the value of examining social determinants of medication adherence through an intersectional lens. Rather than assuming uniform effects of SDoH across populations, future research should employ oversampling strategies and community-based recruitment to better capture underrepresented groups and avoid reproducing inequities in research design. Strengthening diverse patient engagement, expanding community health worker programs, and building culturally responsive adherence interventions remain critical pathways for improving cardiovascular equity.

### Public health and clinical implications

Growing evidence suggests that an exclusive focus on race as a determinant of health disparities can obscure the modifiable structural barriers that actually drive inequities. Our findings support this perspective by illustrating how SDoH—such as education, income, insurance status, and immigration-related barriers—affect populations differently. This underscores the need to move beyond race as a proxy for risk and instead interrogate the structural and social contexts in which racialized inequities arise. Although these findings are derived from a survey-responding subset and should be interpreted cautiously, they still highlight meaningful public health priorities. Efforts to reduce medication non-adherence must account for social and economic inequities that shape patients' ability to engage in long-term cardiovascular prevention (36). Ensuring consistent, affordable, and high-quality access to healthcare is especially important given the potential of preventive therapies such as statins to narrow disparities in cardiovascular disease. Improving system-level responsiveness—through more culturally connected care models, reduced care fragmentation, and policies that alleviate cost burdens—can help create stronger and more equitable care touchpoints (37). Addressing historical and ongoing mistrust in healthcare, particularly among marginalized communities, also remains essential for advancing engagement and adherence (35).

In conclusion, this national study has illuminated the intricate mosaic of factors influencing statin adherence across a racially and ethnically diverse cohort, highlighting the need for multifaceted and culturally sensitive interventions. Indeed, each group's challenges are uniquely shaped by its experiences and social determinants. To improve statin adherence across all populations, interventions must address shared challenges and consider diverse social, cultural, and economic realities. Future research should continue to examine how social conditions and lived experiences influence medication adherence while also improving the representation of diverse populations to better capture diverse patient perspectives. Broader inclusion in clinical research can help address gaps in understanding barriers to adherence and inform more context-sensitive interventions. These findings underscore the need for research and policy strategies that reflect the varying experiences across patient communities.

## Data Availability

The data analyzed in this study are subject to the following licenses/restrictions: The dataset used for this study is part of the All of Us Controlled Tier data, which are accessible only to registered researchers who have completed the required training and obtained approval for use under the program's Data Use Agreement. Access is restricted to ensure participant privacy and data security. Requests to access these datasets should be directed to preston@stanford.edu.
