# Supplementary material for: Racial and ethnic disparities in statin adherence: insights from the All of Us Research Program
Source: Front Cardiovasc Med. 2025 Dec 12;12:1541082. doi: 10.3389/fcvm.2025.1541082 (PMC12740900; doi:10.3389/fcvm.2025.1541082)
Supplement: Supplementary file 1 [file Datasheet1.pdf]

Supplementary Material

eTable 1. Associated codes utilized in data queries in the AoURP
eTable 2. Health Utilization Survey Question Categorizations
eTable 3: Bivariate Analysis of the Survey Cohort According to Percent Days Covered (PDC) of Statin Prescription

eTable 1. Associated codes utilized in data queries in the AoURP

Prescription Name	Type	Query Code
Lovastatin	RxNorm	6472
Pitavastatin	RxNorm	861634
Fluvastatin	RxNorm	41127
Rosuvastatin	RxNorm	301542
Pravastatin	RxNorm	42463
Atorvastatin	RxNorm	83367
Simvastatinn	RxNorm	36567

Regression Variables	Type	Query Code
Age	AoU Source	date_of_birh
Smoking Status	AoU Source	1586182
Educational Attainment	AoU Source	1585940
Income	AoU Source	1585375
Sex at Birth	AoU Source	1585845
Insurance	AoU Source	1585386
Birthplace	AoU Source	1586135
Race/ethnicity	AoU Source	1586140
Employment Status	AoU Source	1585952
Marital Status	AoU Source	1585892
Healthcare Utilization Survey	AoU Source	43528895

Disease Name	Type	Code	Disease Name	Type	Code
Myocardial Infarction	ICD9CM	410	Mild Liver Disease	ICD10	B18
Myocardial Infarction	ICD9CM	412	Mild Liver Disease	ICD10	K73
Congestive Heart Failure	ICD9CM	398.91	Mild Liver Disease	ICD10	K74
Congestive Heart Failure	ICD9CM	402.01	Mild Liver Disease	ICD10	K70.0
Congestive Heart Failure	ICD9CM	402.11	Mild Liver Disease	ICD10	K70.1
Congestive Heart Failure	ICD9CM	402.91	Mild Liver Disease	ICD10	K70.2
Congestive Heart Failure	ICD9CM	404.01	Mild Liver Disease	ICD10	K70.3
Congestive Heart Failure	ICD9CM	404.03	Mild Liver Disease	ICD10	K70.9
Congestive Heart Failure	ICD9CM	404.11	Mild Liver Disease	ICD10	K71.7
Congestive Heart Failure	ICD9CM	404.13	Mild Liver Disease	ICD10	K71.3
Congestive Heart Failure	ICD9CM	404.91	Mild Liver Disease	ICD10	K71.4
Congestive Heart Failure	ICD9CM	404.93	Mild Liver Disease	ICD10	K71.5
Congestive Heart Failure	ICD9CM	425.4	Mild Liver Disease	ICD10	K76.0
Congestive Heart Failure	ICD9CM	425.5	Mild Liver Disease	ICD10	K76.2
Congestive Heart Failure	ICD9CM	425.7	Mild Liver Disease	ICD10	K76.3
Congestive Heart Failure	ICD9CM	425.8	Mild Liver Disease	ICD10	K76.4
Congestive Heart Failure	ICD9CM	425.9	Mild Liver Disease	ICD10	K76.8
Congestive Heart Failure	ICD9CM	428	Mild Liver Disease	ICD10	K76.9
Periphral Vascular Disease	ICD9CM	930	Mild Liver Disease	ICD10	Z94.4
Periphral Vascular Disease	ICD9CM	437.3	Diabetes without complications	ICD10	E10.0

Periphral Vascular Disease	ICD9CM	440	Diabetes without complications	ICD10	E10.1
Periphral Vascular Disease	ICD9CM	441	Diabetes without complications	ICD10	E10.6
Periphral Vascular Disease	ICD9CM	443.1	Diabetes without complications	ICD10	E10.8
Periphral Vascular Disease	ICD9CM	443.2	Diabetes without complications	ICD10	E10.9
Periphral Vascular Disease	ICD9CM	443.8	Diabetes without complications	ICD10	E11.0
Periphral Vascular Disease	ICD9CM	443.9	Diabetes without complications	ICD10	E11.1
Periphral Vascular Disease	ICD9CM	447.1	Diabetes without complications	ICD10	E11.6
Periphral Vascular Disease	ICD9CM	557.1	Diabetes without complications	ICD10	E11.8
Periphral Vascular Disease	ICD9CM	557.9	Diabetes without complications	ICD10	E11.9
Periphral Vascular Disease	ICD9CM	V43.4	Diabetes without complications	ICD10	E12.0
Cerebrovascular Disease	ICD9CM	362.34	Diabetes without complications	ICD10	E12.1
Cerebrovascular Disease	ICD9CM	430	Diabetes without complications	ICD10	E12.6
Cerebrovascular Disease	ICD9CM	431	Diabetes without complications	ICD10	E12.8
Cerebrovascular Disease	ICD9CM	432	Diabetes without complications	ICD10	E12.9
Cerebrovascular Disease	ICD9CM	433	Diabetes without complications	ICD10	E13.0
Cerebrovascular Disease	ICD9CM	434	Diabetes without complications	ICD10	E13.1
Cerebrovascular Disease	ICD9CM	435	Diabetes without complications	ICD10	E13.6
Cerebrovascular Disease	ICD9CM	436	Diabetes without complications	ICD10	E13.8
Cerebrovascular Disease	ICD9CM	437	Diabetes without complications	ICD10	E13.9
Cerebrovascular Disease	ICD9CM	438	Diabetes without complications	ICD10	E14.0
Dementia	ICD9CM	290	Diabetes without complications	ICD10	E14.1
Dementia	ICD9CM	294.1	Diabetes without complications	ICD10	E14.6
Dementia	ICD9CM	331.2	Diabetes without complications	ICD10	E14.8
Chronic Pulmonary Disease	ICD9CM	416.8	Diabetes without complications	ICD10	E14.9
Chronic Pulmonary Disease	ICD9CM	416.9	Diabetes with complications	ICD10	E10.2
Chronic Pulmonary Disease	ICD9CM	490	Diabetes with complications	ICD10	E10.3
Chronic Pulmonary Disease	ICD9CM	491	Diabetes with complications	ICD10	E10.4
Chronic Pulmonary Disease	ICD9CM	492	Diabetes with complications	ICD10	E10.5
Chronic Pulmonary Disease	ICD9CM	493	Diabetes with complications	ICD10	E10.7
Chronic Pulmonary Disease	ICD9CM	494	Diabetes with complications	ICD10	E11.2
Chronic Pulmonary Disease	ICD9CM	495	Diabetes with complications	ICD10	E11.3

Chronic Pulmonary Disease	ICD9CM	496	Diabetes with complications	ICD10	E11.4
Chronic Pulmonary Disease	ICD9CM	500	Diabetes with complications	ICD10	E11.5
Chronic Pulmonary Disease	ICD9CM	501	Diabetes with complications	ICD10	E11.7
Chronic Pulmonary Disease	ICD9CM	502	Diabetes with complications	ICD10	E12.2
Chronic Pulmonary Disease	ICD9CM	503	Diabetes with complications	ICD10	E12.3
Chronic Pulmonary Disease	ICD9CM	504	Diabetes with complications	ICD10	E12.4
Chronic Pulmonary Disease	ICD9CM	505	Diabetes with complications	ICD10	E12.5
Chronic Pulmonary Disease	ICD9CM	506.4	Diabetes with complications	ICD10	E12.7
Chronic Pulmonary Disease	ICD9CM	508.1	Diabetes with complications	ICD10	E13.2
Chronic Pulmonary Disease	ICD9CM	508.8	Diabetes with complications	ICD10	E13.3
Connective Tissue Disease-Rheumatic Disease	ICD9CM	446.5	Diabetes with complications	ICD10	E13.4
Connective Tissue Disease-Rheumatic Disease	ICD9CM	710	Diabetes with complications	ICD10	E13.5
Connective Tissue Disease-Rheumatic Disease	ICD9CM	710.1	Diabetes with complications	ICD10	E13.7
Connective Tissue Disease-Rheumatic Disease	ICD9CM	710.2	Diabetes with complications	ICD10	E14.2
Connective Tissue Disease-Rheumatic Disease	ICD9CM	710.3	Diabetes with complications	ICD10	E14.3
Connective Tissue Disease-Rheumatic Disease	ICD9CM	710.4	Diabetes with complications	ICD10	E14.4
Connective Tissue Disease-Rheumatic Disease	ICD9CM	714	Diabetes with complications	ICD10	E14.5
Connective Tissue Disease-Rheumatic Disease	ICD9CM	714.1	Diabetes with complications	ICD10	E14.7
Connective Tissue Disease-Rheumatic Disease	ICD9CM	714.2	Paraplegia and Hemiplegia	ICD10	G81
Connective Tissue Disease-Rheumatic Disease	ICD9CM	714.8	Paraplegia and Hemiplegia	ICD10	G82
Connective Tissue Disease-Rheumatic Disease	ICD9CM	725	Paraplegia and Hemiplegia	ICD10	G04.1
Peptic Ulcer Disease	ICD9CM	531	Paraplegia and Hemiplegia	ICD10	G11.4
Peptic Ulcer Disease	ICD9CM	532	Paraplegia and Hemiplegia	ICD10	G80.1
Peptic Ulcer Disease	ICD9CM	533	Paraplegia and Hemiplegia	ICD10	G80.2
Peptic Ulcer Disease	ICD9CM	534	Paraplegia and Hemiplegia	ICD10	G83.0
Mild Liver Disease	ICD9CM	702.2	Paraplegia and Hemiplegia	ICD10	G83.1
Mild Liver Disease	ICD9CM	702.3	Paraplegia and Hemiplegia	ICD10	G83.2
Mild Liver Disease	ICD9CM	703.2	Paraplegia and Hemiplegia	ICD10	G83.3
Mild Liver Disease	ICD9CM	703.3	Paraplegia and Hemiplegia	ICD10	G83.4
Mild Liver Disease	ICD9CM	704.4	Paraplegia and Hemiplegia	ICD10	G83.9
Mild Liver Disease	ICD9CM	705.4	Renal Disease	ICD10	N18
Mild Liver Disease	ICD9CM	706	Renal Disease	ICD10	N19

Mild Liver Disease	ICD9CM	709	Renal Disease	ICD10	N05.2
Mild Liver Disease	ICD9CM	570	Renal Disease	ICD10	N05.3
Mild Liver Disease	ICD9CM	571	Renal Disease	ICD10	N05.4
Mild Liver Disease	ICD9CM	573.3	Renal Disease	ICD10	N05.5
Mild Liver Disease	ICD9CM	573.4	Renal Disease	ICD10	N05.6
Mild Liver Disease	ICD9CM	573.8	Renal Disease	ICD10	N05.7
Mild Liver Disease	ICD9CM	573.9	Renal Disease	ICD10	N25.0
Mild Liver Disease	ICD9CM	V42.7	Renal Disease	ICD10	I12.0
Diabetes without complications	ICD9CM	250	Renal Disease	ICD10	I13.1
Diabetes without complications	ICD9CM	250.1	Renal Disease	ICD10	N03.2
Diabetes without complications	ICD9CM	250.2	Renal Disease	ICD10	N03.3
Diabetes without complications	ICD9CM	250.3	Renal Disease	ICD10	N03.4
Diabetes without complications	ICD9CM	250.8	Renal Disease	ICD10	N03.5
Diabetes without complications	ICD9CM	250.9	Renal Disease	ICD10	N03.6
Diabetes with complications	ICD9CM	250.4	Renal Disease	ICD10	N03.7
Diabetes with complications	ICD9CM	250.5	Renal Disease	ICD10	Z49.0
Diabetes with complications	ICD9CM	250.6	Renal Disease	ICD10	Z49.1
Diabetes with complications	ICD9CM	250.7	Renal Disease	ICD10	Z49.2
Paraplegia and Hemiplegia	ICD9CM	334.1	Renal Disease	ICD10	Z94.0
Paraplegia and Hemiplegia	ICD9CM	342	Renal Disease	ICD10	Z99.2
Paraplegia and Hemiplegia	ICD9CM	343	Cancer	ICD10	C00
Paraplegia and Hemiplegia	ICD9CM	344	Cancer	ICD10	C01
Paraplegia and Hemiplegia	ICD9CM	344.1	Cancer	ICD10	C02
Paraplegia and Hemiplegia	ICD9CM	344.2	Cancer	ICD10	C03
Paraplegia and Hemiplegia	ICD9CM	344.3	Cancer	ICD10	C04
Paraplegia and Hemiplegia	ICD9CM	344.4	Cancer	ICD10	C05
Paraplegia and Hemiplegia	ICD9CM	344.5	Cancer	ICD10	C06
Paraplegia and Hemiplegia	ICD9CM	344.6	Cancer	ICD10	C07
Paraplegia and Hemiplegia	ICD9CM	344.9	Cancer	ICD10	C08
Renal Disease	ICD9CM	403.01	Cancer	ICD10	C09
Renal Disease	ICD9CM	403.11	Cancer	ICD10	C10
Renal Disease	ICD9CM	403.91	Cancer	ICD10	C11
Renal Disease	ICD9CM	404.02	Cancer	ICD10	C12
Renal Disease	ICD9CM	404.03	Cancer	ICD10	C13
Renal Disease	ICD9CM	404.12	Cancer	ICD10	C14
Renal Disease	ICD9CM	404.13	Cancer	ICD10	C15
Renal Disease	ICD9CM	404.92	Cancer	ICD10	C16
Renal Disease	ICD9CM	404.93	Cancer	ICD10	C17
Renal Disease	ICD9CM	582	Cancer	ICD10	C18
Renal Disease	ICD9CM	583	Cancer	ICD10	C19
Renal Disease	ICD9CM	583.1	Cancer	ICD10	C20
Renal Disease	ICD9CM	583.2	Cancer	ICD10	C21
Renal Disease	ICD9CM	583.4	Cancer	ICD10	C22
Renal Disease	ICD9CM	583.6	Cancer	ICD10	C23
Renal Disease	ICD9CM	583.7	Cancer	ICD10	C24
Renal Disease	ICD9CM	585	Cancer	ICD10	C25
Renal Disease	ICD9CM	586	Cancer	ICD10	C26
Renal Disease	ICD9CM	588	Cancer	ICD10	C30
Renal Disease	ICD9CM	V42.0	Cancer	ICD10	C31
Renal Disease	ICD9CM	V45.1	Cancer	ICD10	C32
Renal Disease	ICD9CM	V56	Cancer	ICD10	C33
Cancer	ICD9CM	140	Cancer	ICD10	C34
Cancer	ICD9CM	141	Cancer	ICD10	C37
Cancer	ICD9CM	142	Cancer	ICD10	C38
Cancer	ICD9CM	143	Cancer	ICD10	C39
Cancer	ICD9CM	144	Cancer	ICD10	C40

Cancer	ICD9CM	145	Cancer	ICD10	C41
Cancer	ICD9CM	146	Cancer	ICD10	C43
Cancer	ICD9CM	147	Cancer	ICD10	C45
Cancer	ICD9CM	148	Cancer	ICD10	C46
Cancer	ICD9CM	149	Cancer	ICD10	C47
Cancer	ICD9CM	150	Cancer	ICD10	C48
Cancer	ICD9CM	151	Cancer	ICD10	C49
Cancer	ICD9CM	152	Cancer	ICD10	C50
Cancer	ICD9CM	153	Cancer	ICD10	C51
Cancer	ICD9CM	154	Cancer	ICD10	C52
Cancer	ICD9CM	155	Cancer	ICD10	C53
Cancer	ICD9CM	156	Cancer	ICD10	C54
Cancer	ICD9CM	157	Cancer	ICD10	C55
Cancer	ICD9CM	158	Cancer	ICD10	C56
Cancer	ICD9CM	159	Cancer	ICD10	C57
Cancer	ICD9CM	160	Cancer	ICD10	C58
Cancer	ICD9CM	161	Cancer	ICD10	C60
Cancer	ICD9CM	162	Cancer	ICD10	C61
Cancer	ICD9CM	163	Cancer	ICD10	C62
Cancer	ICD9CM	164	Cancer	ICD10	C63
Cancer	ICD9CM	165	Cancer	ICD10	C64
Cancer	ICD9CM	170	Cancer	ICD10	C65
Cancer	ICD9CM	171	Cancer	ICD10	C66
Cancer	ICD9CM	172	Cancer	ICD10	C67
Cancer	ICD9CM	174	Cancer	ICD10	C68
Cancer	ICD9CM	175	Cancer	ICD10	C69
Cancer	ICD9CM	176	Cancer	ICD10	C70
Cancer	ICD9CM	179	Cancer	ICD10	C71
Cancer	ICD9CM	180	Cancer	ICD10	C72
Cancer	ICD9CM	181	Cancer	ICD10	C73
Cancer	ICD9CM	182	Cancer	ICD10	C74
Cancer	ICD9CM	183	Cancer	ICD10	C75
Cancer	ICD9CM	184	Cancer	ICD10	C76
Cancer	ICD9CM	185	Cancer	ICD10	C81
Cancer	ICD9CM	186	Cancer	ICD10	C82
Cancer	ICD9CM	187	Cancer	ICD10	C83
Cancer	ICD9CM	188	Cancer	ICD10	C84
Cancer	ICD9CM	189	Cancer	ICD10	C85
Cancer	ICD9CM	190	Cancer	ICD10	C88
Cancer	ICD9CM	191	Cancer	ICD10	C90
Cancer	ICD9CM	192	Cancer	ICD10	C91
Cancer	ICD9CM	193	Cancer	ICD10	C92
Cancer	ICD9CM	194	Cancer	ICD10	C93
Cancer	ICD9CM	195	Cancer	ICD10	C94
Cancer	ICD9CM	200	Cancer	ICD10	C95
Cancer	ICD9CM	201	Cancer	ICD10	C96
Cancer	ICD9CM	202	Cancer	ICD10	C97
Cancer	ICD9CM	203	Moderate or Severe Liver Disease	ICD10	K70.4
Cancer	ICD9CM	204	Moderate or Severe Liver Disease	ICD10	K71.1
Cancer	ICD9CM	205	Moderate or Severe Liver Disease	ICD10	K72.1
Cancer	ICD9CM	206	Moderate or Severe Liver Disease	ICD10	K72.9
Cancer	ICD9CM	207	Moderate or Severe Liver Disease	ICD10	K76.5
Cancer	ICD9CM	208	Moderate or Severe Liver Disease	ICD10	K76.6
Cancer	ICD9CM	238.6	Moderate or Severe Liver Disease	ICD10	K76.7

Moderate or Severe Liver Disease	ICD9CM	456	Moderate or Severe Liver Disease	ICD10	I85.0
Moderate or Severe Liver Disease	ICD9CM	456.1	Moderate or Severe Liver Disease	ICD10	I85.9
Moderate or Severe Liver Disease	ICD9CM	456.2	Moderate or Severe Liver Disease	ICD10	I86.4
Moderate or Severe Liver Disease	ICD9CM	572.2	Moderate or Severe Liver Disease	ICD10	I98.2
Moderate or Severe Liver Disease	ICD9CM	572.3	Metastatic Carcinoma	ICD10	C77
Moderate or Severe Liver Disease	ICD9CM	572.4	Metastatic Carcinoma	ICD10	C78
Moderate or Severe Liver Disease	ICD9CM	572.8	Metastatic Carcinoma	ICD10	C79
Metastatic Carcinoma	ICD9CM	196	Metastatic Carcinoma	ICD10	C80
Metastatic Carcinoma	ICD9CM	197	AIDS/HIV	ICD10	B20
Metastatic Carcinoma	ICD9CM	198	AIDS/HIV	ICD10	B21
Metastatic Carcinoma	ICD9CM	199	AIDS/HIV	ICD10	B22
AIDS/HIV	ICD9CM	42	AIDS/HIV	ICD10	B24
AIDS/HIV	ICD9CM	43	Cerebrovascular Disease	ICD10	G45
AIDS/HIV	ICD9CM	44	Cerebrovascular Disease	ICD10	G46
Myocardial Infarction	ICD10	I21	Cerebrovascular Disease	ICD10	I60
Myocardial Infarction	ICD10	I22	Cerebrovascular Disease	ICD10	I61
Myocardial Infarction	ICD10	I25.2	Cerebrovascular Disease	ICD10	I62
Congestive Heart Failure	ICD10	I43	Cerebrovascular Disease	ICD10	I63
Congestive Heart Failure	ICD10	I50	Cerebrovascular Disease	ICD10	I64
Congestive Heart Failure	ICD10	I09.9	Cerebrovascular Disease	ICD10	I65
Congestive Heart Failure	ICD10	I11.0	Cerebrovascular Disease	ICD10	I66
Congestive Heart Failure	ICD10	I13.0	Cerebrovascular Disease	ICD10	I67
Congestive Heart Failure	ICD10	I13.2	Cerebrovascular Disease	ICD10	I68
Congestive Heart Failure	ICD10	I25.5	Cerebrovascular Disease	ICD10	I69
Congestive Heart Failure	ICD10	I42.0	Cerebrovascular Disease	ICD10	H34.0
Congestive Heart Failure	ICD10	I42.5	Dementia	ICD10	F00
Congestive Heart Failure	ICD10	I42.6	Dementia	ICD10	F01
Congestive Heart Failure	ICD10	I42.7	Dementia	ICD10	F02
Congestive Heart Failure	ICD10	I42.8	Dementia	ICD10	F03
Congestive Heart Failure	ICD10	I42.9	Dementia	ICD10	G30
Congestive Heart Failure	ICD10	P29.0	Dementia	ICD10	F05.1
Periphral Vascular Disease	ICD10	I70	Dementia	ICD10	G31.1
Periphral Vascular Disease	ICD10	I71	Chronic Pulmonary Disease	ICD10	J40
Periphral Vascular Disease	ICD10	I73.1	Chronic Pulmonary Disease	ICD10	J41
Periphral Vascular Disease	ICD10	I73.8	Chronic Pulmonary Disease	ICD10	J42
Periphral Vascular Disease	ICD10	I73.9	Chronic Pulmonary Disease	ICD10	J43

Periphral Vascular Disease	ICD10	I77.1	Chronic Pulmonary Disease	ICD10	J44
Periphral Vascular Disease	ICD10	I79.0	Chronic Pulmonary Disease	ICD10	J45
Periphral Vascular Disease	ICD10	I79.2	Chronic Pulmonary Disease	ICD10	J46
Periphral Vascular Disease	ICD10	K55.1	Chronic Pulmonary Disease	ICD10	J47
Periphral Vascular Disease	ICD10	K55.8	Chronic Pulmonary Disease	ICD10	J60
Periphral Vascular Disease	ICD10	K55.9	Chronic Pulmonary Disease	ICD10	J61
Periphral Vascular Disease	ICD10	Z95.8	Chronic Pulmonary Disease	ICD10	J62
Periphral Vascular Disease	ICD10	Z95.9	Chronic Pulmonary Disease	ICD10	J63
Connective Tissue Disease-Rheumatic Disease	ICD10	M05	Chronic Pulmonary Disease	ICD10	J64
Connective Tissue Disease-Rheumatic Disease	ICD10	M32	Chronic Pulmonary Disease	ICD10	J65
Connective Tissue Disease-Rheumatic Disease	ICD10	M33	Chronic Pulmonary Disease	ICD10	J66
Connective Tissue Disease-Rheumatic Disease	ICD10	M34	Chronic Pulmonary Disease	ICD10	J67.I278
Connective Tissue Disease-Rheumatic Disease	ICD10	M06	Chronic Pulmonary Disease	ICD10	I27.9
Connective Tissue Disease-Rheumatic Disease	ICD10	M31.5	Chronic Pulmonary Disease	ICD10	J68.4
Connective Tissue Disease-Rheumatic Disease	ICD10	M35.1	Chronic Pulmonary Disease	ICD10	J70.1
Connective Tissue Disease-Rheumatic Disease	ICD10	M35.3	Chronic Pulmonary Disease	ICD10	J70.3
Connective Tissue Disease-Rheumatic Disease	ICD10	M36.0			
Peptic Ulcer Disease	ICD10	K25			
Peptic Ulcer Disease	ICD10	K26			
Peptic Ulcer Disease	ICD10	K27			
Peptic Ulcer Disease	ICD10	K28			

eTable 2. Health Utilization Survey Question Categorizations

Category Name	Question found in Survey	Number of respondents
Mutual Relationship with Provider	How often did your doctors or health care providers ask for your opinions or beliefs about your medical care or treatment? For example, what kind of tests, procedures, or medications you prefer.	7,498
	How often did your doctors or health care providers tell or give you information about your health and health care that was easy to understand?	7,585
	How often were you treated with respect by your doctors or health care providers?	7,604
Delayed Care Due to Lack of Diversity	How often have you either delayed or not gone to see doctors or health care providers because they were different from you in any of these ways?	7,528
Financial Barrier	If you get sick or have an accident, how worried are you that you will be able to pay your medical bills? Are you very worried, somewhat worried, or not at all worried?	7,572
	DURING THE PAST 12 MONTHS, were any of the following true for you:	
	You used alternative therapies to save money	6,973
	You bought prescription drugs from another country to save money	7,054
	You delayed filling a prescription to save money	7,502
	You asked your doctor for a lower cost medication to save money	7,213
	You skipped medication doses to save money	7,751
	You took less medicine to save money	7,786
Provider Inaccessibility	Is there a place that you USUALLY go to when you are sick or need advice about your health?	7,633
	About how long has it been since you last saw or talked to a doctor or other health care provider about your own health?	7,601
Insurance Barrier	DURING THE PAST 12 MONTHS, were you told by a health care provider or doctor’s office that they did not accept your health care coverage?	7,689

	Have you delayed getting care for any of the following reasons in the PAST 12 MONTHS:	
	Couldn’t afford the copay.	6,534
	Your deductible was too high/or could not afford the deductible.	6,470
	You had to pay out of pocket for some or all of the procedure.	6,474
	In regard to your health insurance or health care coverage, how does it compare to a year ago? Is it better, worse, or about the same?	7,631
	Have you delayed getting care for any of the following reasons in the PAST 12 MONTHS:	
Patient Anxiety	You were nervous about seeing your healthcare provider.	7,252
Rural Barrier	Have you delayed getting care for any of the following reasons in the PAST 12 MONTHS: You live in a rural area where distance to the health care provider is too far.	7,501
Social Dependency	Have you delayed getting care for any of the following reasons in the PAST 12 MONTHS: Couldn’t get childcare.	6,549
Transportation Barrier	Have you delayed getting care for any of the following reasons in the PAST 12 MONTHS: Didn’t have transportation.	7,628
Employment Barrier	Have you delayed getting care for any of the following reasons in the PAST 12 MONTHS: Couldn’t get time off work.	6,952

eTable 3: Bivariate Analysis of the Survey Cohort According to Percent Days Covered (PDC) of Statin Prescription

	Survey Cohort		
	**PDC < 0.80 N= 4734**	**PDC => 0.80 N= 3052**	**Odds Ratio (95% CI)**
**Age (mean (SD))**	64.33 (11.20)	65.85 (10.09)	1.01 (1.01, 1.02)
**Sex at Birth – Female (ref: Male) (%)**	2416 (51.0)	1493 (48.9)	0.92 (0.84, 1.01))
**Race (%)**			
**Non-Hispanic Black**	650 (13.7)	407 (13.3)	0.91 (0.8, 1.04)
**Hispanic**	330 (7.0)	62 (2.0)	0.27 (0.21, 0.36)
**Non-Hispanic White**	3754 (79.3)	2583 (84.6)	Reference
**Insurance (%)**			
**Medicaid**	1826 (38.6)	1239 (40.6)	1.11 (0.96, 1.29)
**Medicare**	722 (15.3)	455 (14.9)	1.2 (1.07, 1.34)
**Other**	290 (6.1)	169 (5.5)	1.03 (0.83, 1.27)
**Private**	1330 (28.1)	754 (24.7)	
**VA**	566 (12.0)	435 (14.3)	1.36 (1.16, 1.58)
**Missing**	-	-	-
**Education (%)**			
**Less than secondary**	77 (1.6)	29 (1.0)	0.58 (0.37, 0.88)
**Secondary**	821 (17.3)	527 (17.3)	0.99 (0.87, 1.11)
**Post-Secondary**	3836 (81.0)	2496 (81.8)	Reference
**Missing**	-	-	-
**Income (%)**			
**Low <$ 35K**	1564 (33.0)	980 (32.1)	1.02 (0.91, 1.14)
**Middle $35K – $75K**	1410 (29.8)	992 (32.5)	1.15 (1.03, 1.28)
**High >$ 75k**	1760 (37.2)	1080 (35.4)	Reference
**Missing**			
**Foreign Born (%)**	311 (6.6)	91 (3.0)	0.44 (0.34, 0.55)
**Not Employed (%)**	3037 (64.2)	2103 (68.9)	1.24 (1.12, 1.36)
**Marital Status (%)**			
**Married/Widowed**	2793 (59.0)	1805 (59.1)	1 (0.87, 1.15)
**Separated/Divorce**	1356 (28.6)	869 (28.5)	0.99 (0.85, 1.16)
**Single**	585 (12.4)	378 (12.4)	Reference
**Missing**			
**Smoking (%)**			
**Current**	491 (10.4)	280 (9.2)	0.91 (0.77, 1.06)
**Former**	1798 (38.0)	1234 (40.4)	1.09 (0.99, 1.2)
**Never**	2445 (51.6)	1538 (50.4)	
**Missing**	-	-	-
**CCI High (%)**	3543 (74.8)	2446 (80.1)	1.36 (1.22, 1.52)
**Serum Creatinine (mean (SD))**	2.5 (3.6)	1.9 (2.9)	0.94 (0.93, 0.96)
**SBP (mean (SD))**	131.0 (16.6)	131.2 (16.4)	1 (1,1)
**HDL (mean (SD))**	38.0 (16.4)	38.5 (14.9	1 (1, 1)
**LDL (mean (SD))**	90.7 (36.4)	81.0 (30.7)	0.99 (0.99,0.99)
**Total Chol (mean(SD))**	177.5 (45.0)	167.6 (38.9)	0.99 (0.99, 1)
**DBP (mean (SD))**	77.7 (10.3)	76.9 (10.0)	0.99 (0.99,0.99)
**BMI (mean (SD))**	31.7 (7.2)	31.8 (7.0)	0.99 (0.99,0.99)
**MI (%)**	474 (10.0)	343 (11.2)	1.14 (0.98, 1.32)
**CHF (%)**	616 (13.0)	428 (14.0)	1.09 (0.95, 1.24)
**Liver Disease (%)**	772 (16.3)	518 (17.0)	1.05 (0.93, 1.18)
**Any Diabetes (%)**	1630 (34.4)	1171 (38.4)	1.19 (1.08, 1.3)

Continuous variables are reported as means with standard deviations, and categorical variables are reported as percentages. Unadjusted odds ratios (95%CI) are reported for each variable.

† Other race category includes American Indian/Alaskan native, Middle Eastern, Asian, Native Hawaiian/ Pacific Islander.

Abbreviations: PDC: Percent Days Covered, CI: Confidence Interval, SD: Standard Deviation, CCI: Charlson Comorbidity Index, SBP: Systolic Blood Pressure, HDL: High Density Lipoprotein, LDL: Low Density Lipoprotein, Chol: Cholesterol, DBP: Diastolic Blood Pressure, BMI: Body Mass Index, MI: Myocardial Infarction, CHF: Congestive Heart Failure
